# Supplementary material for: The Prisoner’s Dilemma paradigm provides a neurobiological framework for the social decision cascade
Source: PLoS One. 2021 Mar 18;16(3):e0248006. doi: 10.1371/journal.pone.0248006 (PMC7971531; doi:10.1371/journal.pone.0248006)
Supplement: S1 File — (DOCX) [file pone.0248006.s001.docx]

At the end of the study session, in accordance with guidelines for ethically appropriate authorized deception, participants were debriefed about the deception involved in the task and the motivation for its use. They were informed at consent that during the study protocol they would be given misleading or inaccurate information, but they were not told when this would occur. During post-game debriefing, participants were read a standardized statement that describes how they had been deceived and explained that deception was necessary to ensure that they experienced the game as a “real” interaction with another person. After the researcher explained the deception process and rationale, participants were asked if they had been deceived and encouraged to express any concerns that they had about the deception. No participants expressed concerns and all participants were successfully deceived.
