# Supplementary material for: The Prisoner’s Dilemma paradigm provides a neurobiological framework for the social decision cascade
Source: PLoS One. 2021 Mar 18;16(3):e0248006. doi: 10.1371/journal.pone.0248006 (PMC7971531; doi:10.1371/journal.pone.0248006)
Supplement: S2 File — (DOCX) [file pone.0248006.s002.docx]

The computerized co-player, which operates according to an algorithm based on human patterns of play (McClure et al., 2007) always cooperates during the first round of a game and always defects during the final two rounds of the game. During the other rounds of the game, the computer selects a “choice” based on the participants pattern of decisions in the prior two rounds. A pattern of defection in the prior two rounds increases the likelihood of computer defection, while a pattern of cooperation in the prior two rounds increases the likelihood of computer cooperation. The algorithm additionally establishes a 50% likelihood that the computer will defect after four consecutive rounds of mutual cooperation. We specified this behavior because prior research has shown that in an iterated game, players engage in mutual cooperation for the majority of the task (Rilling et al., [2002](https://link.springer.com/article/10.1007%2Fs10802-007-9113-8#CR46)) and such a pattern of play would prevent participants from experiencing perceived betrayal (DC trials) in a suitable number of rounds for subsequent statistical analysis. A complete breakdown of algorithm behavior is presented below.

1. Round 1: 100%
2. Round 2:
   1. If Round 1 outcome was CC, then 93%
   2. If Round 1 outcome was DC, then 36%
3. Rounds 3-10:
   1. If outcome of 1^st^ 2 rounds was CCCC: 92%
   2. If outcome of prior 2 rounds was CDCC: 86%
   3. If outcome of prior 2 rounds was DCCC: 78%
   4. If outcome of prior 2 rounds was DDCC: 50%
   5. If outcome of prior 2 rounds was CCCD: 58%
   6. If outcome of prior 2 rounds was CDCD: 0%
   7. If outcome of prior 2 rounds was DCCD: 33%
   8. If outcome of prior 2 rounds was DDCD: 33%
   9. If outcome of prior 2 rounds was CCDC: 86%
   10. If outcome of prior 2 rounds was CDDC: 80%
   11. If outcome of prior 2 rounds was DCDC: 33%
   12. If outcome of prior 2 rounds was DDDC: 20%
   13. If outcome of prior 2 rounds was CCDD: 50%
   14. If outcome of prior 2 rounds was CDDD: 38%
   15. If outcome of prior 2 rounds was DCDD: 50%
   16. If outcome of prior 2 rounds was DDDD: 43%
4. Rounds 11-18:
   1. If outcome of prior 2 rounds was CCCC: 92%
   2. If outcome of prior 2 rounds was CDCC: 90%
   3. If outcome of prior 2 rounds was DCCC: 100%
   4. If outcome of prior 2 rounds was DDCC: 60%
   5. If outcome of prior 2 rounds was CCCD: 13%
   6. If outcome of prior 2 rounds was CDCD: 20%
   7. If outcome of prior 2 rounds was DCCD: 67%
   8. If outcome of prior 2 rounds was DDCD: 33%
   9. If outcome of prior 2 rounds was CCDC: 83%
   10. If outcome of prior 2 rounds was CDDC: 63%
   11. If outcome of prior 2 rounds was DCDC: 0%
   12. If outcome of prior 2 rounds was DDDC: 33%
   13. If outcome of prior 2 rounds was CCDD: 33%
   14. If outcome of prior 2 rounds was CDDD: 8%
   15. If outcome of prior 2 rounds was DCDD: 50%
   16. If outcome of prior 2 rounds was DDDD: 25%
5. Rounds 19-20: 0%
