# Supplementary material for: The Prisoner’s Dilemma paradigm provides a neurobiological framework for the social decision cascade
Source: PLoS One. 2021 Mar 18;16(3):e0248006. doi: 10.1371/journal.pone.0248006 (PMC7971531; doi:10.1371/journal.pone.0248006)
Supplement: S3 File — (DOCX) [file pone.0248006.s003.docx]

There is no explicit jittered ISI between the decision, anticipation and the feedback phases of our GLM design matrix. However, the way that we constructed our regressors embedded, in effect, an implicit ISI. Participants all had a full 6 seconds to make a decision; each decision for each participant occurred at a different point in this 6-second window. Thus, the time remaining between the registration of a decision and the presentation of feedback always varied between 0 and 6 seconds, as did the onset of the anticipation phase. The implicit jittering that this task characteristic introduced, together with the original explicit ISI (3, 6 or 9 sec) between decision and feedback phases, continually shifts the onset of the anticipation phase, as well as that of the feedback phase, from round to round. In other words, our design matrix ensures that BOLD activity is sampled at unique, varying time points from phase to phase.
