# Supplementary material for: The Prisoner’s Dilemma paradigm provides a neurobiological framework for the social decision cascade
Source: PLoS One. 2021 Mar 18;16(3):e0248006. doi: 10.1371/journal.pone.0248006 (PMC7971531; doi:10.1371/journal.pone.0248006)
Supplement: S4 File — (DOCX) [file pone.0248006.s004.docx]

**Computer Game Neuroimaging Analysis**

*Decision Phase*

During the decision to cooperate, significant activity was elicited in the right dlPFC and right vlPFC along with the superior and inferior parietal lobules. The decision to cooperate was not significantly associated with activity in the dmPFC, aMCC, TPJ, precuneus or the anterior insula, all regions that were engaged during human gameplay. Full results are listed in Table 1.

During the decision to defect, patterns of activation more closely corresponded with what was seen during human gameplay. The right dlPFC, left vlPFC and the aMCC encompassed prefrontal cortical activation. Activity in the parietal lobes included the TPJ, precuneus, inferior and superior parietal lobules. Finally, significant bilateral hippocampi was detected (see Table 2).

*Anticipation*

Individual voxels during this phase did not survive FWE correction in a whole brain voxel-wise analysis. In a cluster-wise thresholded analysis, during anticipation following cooperation, only two clusters located in the middle occipital lobe were engaged during this phase (see Table 3).

During anticipation following defection, results once again mirrored the pattern of activity observed during human gameplay. Regions of cluster-wise thresholded activation were detected in the dmPFC, bilateral vlPFC, the bilateral TPJ, right superior parietal lobule and the right midde temporal gyrus (see Table 3).

*Feedback*

See Table 4 for detailed results. Across all four feedback conditions, distinct patterns of voxel-wise thresholded activation emerged between: 1) reciprocated feedback and feedback following co-player cooperation and 2) unreciprocated feedback and feedback following co-player defection. Reciprocated feedback and feedback following co-player cooperation was characterized by activity in the right dlPFC and the inferior parietal lobules. In comparison, unreciprocated feedback and feedback following co-player defection elicited significant activity in a more distributed and diverse group of regions including the right dlPFC, vlPFC, dmPFC/aMCC, TPJ, superior parietal lobule and the precuneus. Notably, in contrast to the processing of feedback during human gameplay, the computer feedback conditions failed to elicit significant activity in the anterior insula, hippocampi, and temporal poles, regions integral to aversive response and socioemotional contextualization of events.

***Computer Game Direct Contrasts Analysis***

The results of all within-phase contrasts were cluster-wise thresholded while the results of the between phase contrasts survived whole brain voxel-wise thresholding and FWE correction. A direct contrast between decision-making conditions revealed limited activity in the lingual gyrus when participants chose to defect versus cooperate. No suprathreshold voxels were detected in the direct contrast between anticipation conditions during computer gameplay. However, during the processing of feedback, the direct contrast revealed significantly heightened activity in the posterior midcingulate and the postcentral gyrus when processing reciprocated versus unreciprocated feedback. Significantly activity was elicited in the dmPFC, ACC, mOFC and cerebellum when processing unreciprocated versus reciprocated feedback. No suprathreshold voxels were detected in the direct contrast between feedback following co-player cooperation and co-defection (see table 5 for all direct comparison results during computer gameplay).

Direct contrasts between phases resulted in significant voxel-wise thresholded results for the same contrasts identified in the human game analysis: Decision>Feedback, Decision>Anticipation and Feedback>Anticipation. These contrasts all detected significant activity in the vlPFC, inferior parietal lobules, superior parietal lobules, and hippocampi. Contrast specific differences included significant activity in the bilateral dlPFC, left temporal pole and putamen during the Decision>Feedback and Decision>Anticipation contrasts. TPJ activity was only found during the Decision>Anticipation and Feedback>Anticipation contrasts. Finally, the aMCC and the precuneus was significantly recruited only within the Feedback>Anticipation contrast.

Finally, direct comparison contrast between BOLD activity during human gameplay versus BOLD activity during computer gameplay within all phases of the task produced no significant results within any phase except for feedback during co-player defection. A cluster-wise thresholded analysis of activity during the processing of feedback during co-player defection revealed that a cluster of activity in the left anterior insula was more significantly activated when receiving this form of feedback from a human than a computer co-player. A decomposition of this analysis was ran to determine if unreciprocated cooperation (CD) or mutual defection (DD) was driving this result. This analysis showed that unreciprocated cooperation, not mutual defection was associated with heightened activity in the left anterior insula during human gameplay in comparison to computer gameplay (see table 4 for the results of the direct contrasts between co-player games).
