# Supplementary material for: The Prisoner’s Dilemma paradigm provides a neurobiological framework for the social decision cascade
Source: PLoS One. 2021 Mar 18;16(3):e0248006. doi: 10.1371/journal.pone.0248006 (PMC7971531; doi:10.1371/journal.pone.0248006)
Supplement: S5 File — (DOCX) [file pone.0248006.s005.docx]

**Neuroimaging Analysis of the First Human PD Game vs. Computer Game**

All of the original baseline and direct contrast analyses from the primary manuscript were re-run with rounds from the second human PD game removed to balance the comparison between human and computer conditions by equalizing the statistical power of the analyses. An overlay between both the first PD game and computer game reveal overwhelmingly similarities in spatial extent when comparing the first game analysis to the combined human analysis. Overall, the number of peak voxels were slightly reduced in the baseline contrasts and moderately reduced in the within-phase contrasts which was expected given the disproportionate behavioral trends of some participants when they initially play the game (adopting predominantly cooperation or defection strategies). Finally, a rerun of the human vs computer direct contrast analysis revealed that a significant cluster in the left anterior insula was still detected when comparing response to co-player defection and unreciprocated cooperation during human versus computer play, showing that the increase in power for the human contrasts in the original analyses only provided marginal benefits to the overall analysis.
