# Supplementary figures and images for: The Prisoner’s Dilemma paradigm provides a neurobiological framework for the social decision cascade
Source: PLoS One. 2021 Mar 18;16(3):e0248006. doi: 10.1371/journal.pone.0248006 (PMC7971531; doi:10.1371/journal.pone.0248006)

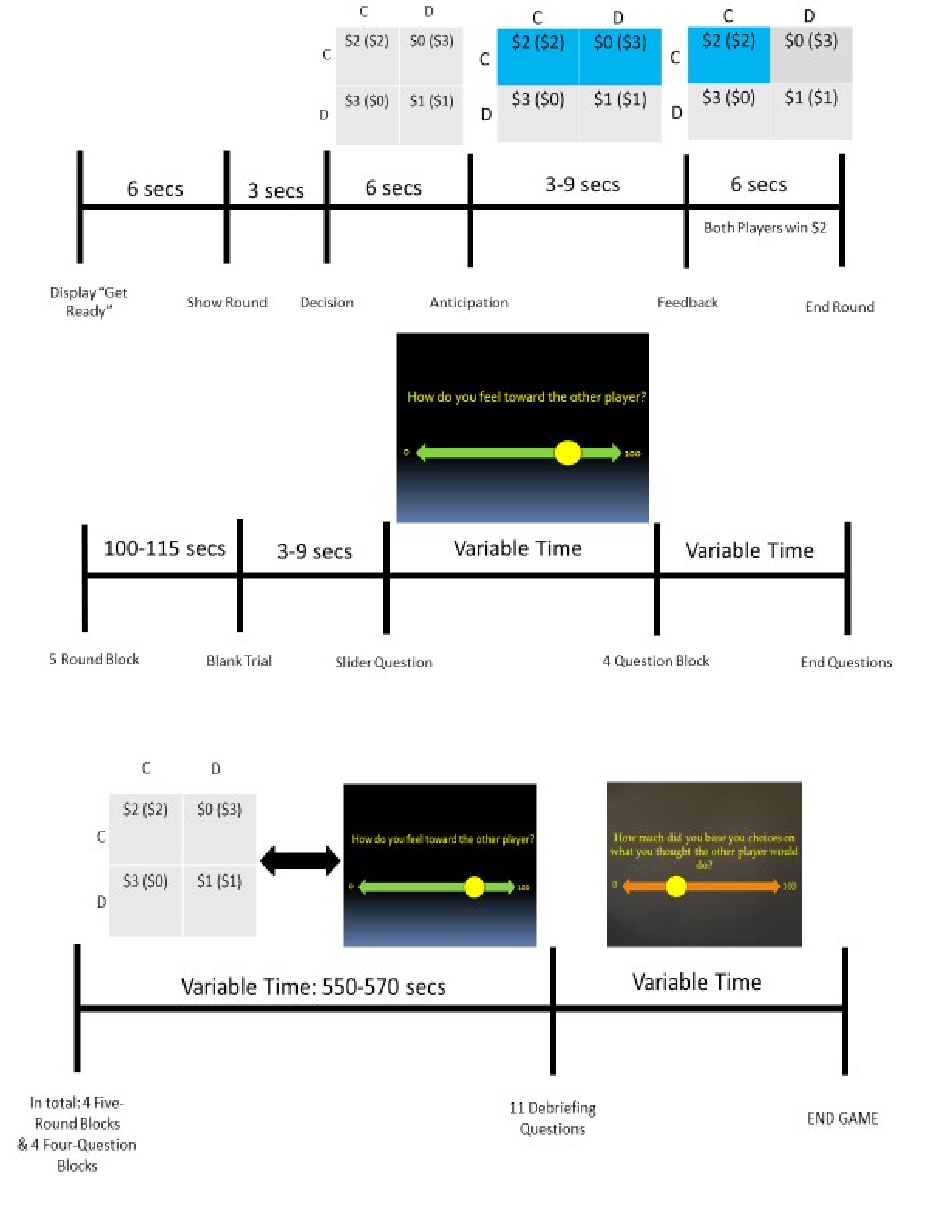

Supplement: S1 Fig — The chart progresses from left-to-right, top-to-bottom. The first section details the timing of one round. The round always begins at “show round”, “Get Ready” only preceded the first round. The second section details the timing of one block, which includes 5 trials and a set of four assessment questions. The final section details the timing of the entire four block run which concludes with 11 debriefing questions to end the game. (TIF) [file pone.0248006.s006.tif]

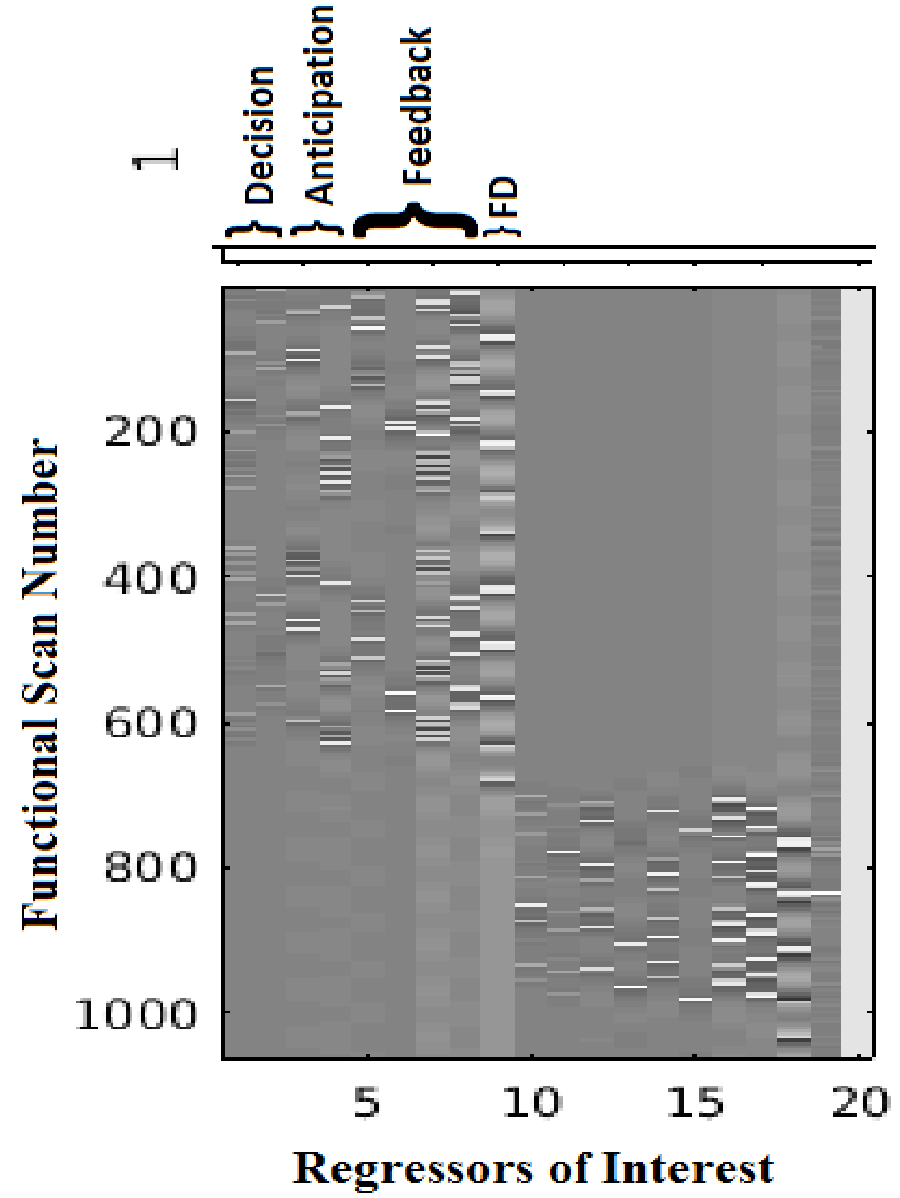

Supplement: S2 Fig — (TIF) [file pone.0248006.s007.tif]

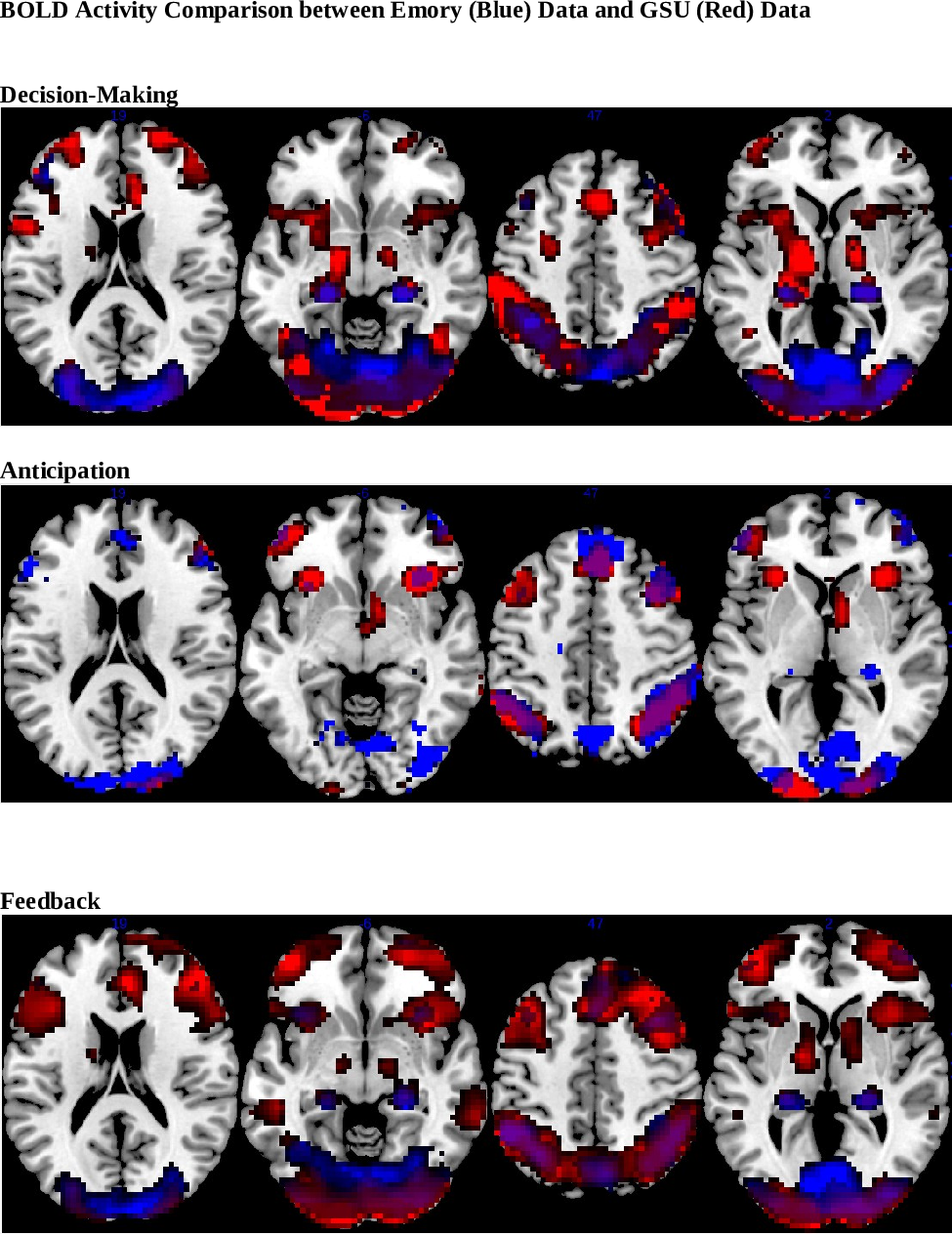

Supplement: S3 Fig — There were no significant differences in patterns of activation between the datasets. Decision: (GSU, t(16) = 3.69, p < .001 uncorrected; Emory, t(13) = 3.85, p < .001 uncorrected). Anticipation: (GSU, t(16) = 1.80, p < .05 uncorrected; Emory, t(13) = 1.75, p < .05 uncorrected). Feedback: (GSU, t(16) = 3.89, p < .001 uncorrected; Emory, t(13) = 3.85, p < .001 uncorrected). (TIF) [file pone.0248006.s008.tif]

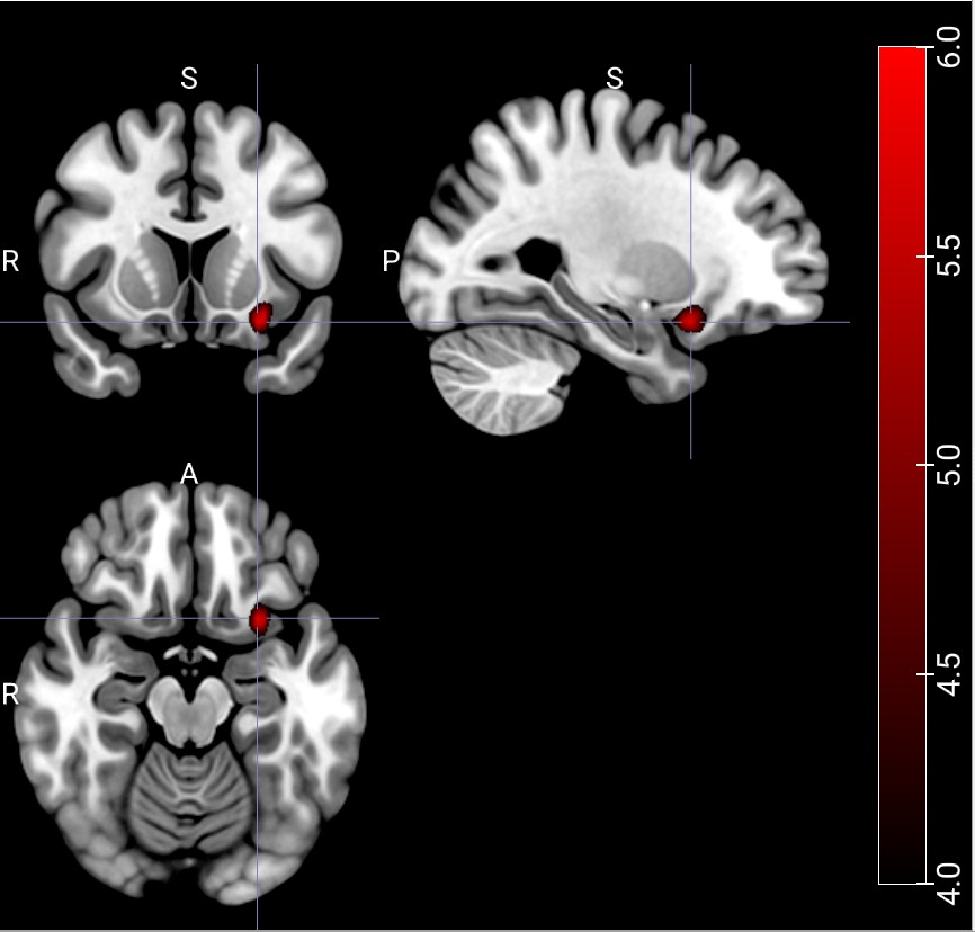

Supplement: S4 Fig — (TIF) [file pone.0248006.s009.tif]
