# Supplementary material for: The Prisoner’s Dilemma paradigm provides a neurobiological framework for the social decision cascade
Source: PLoS One. 2021 Mar 18;16(3):e0248006. doi: 10.1371/journal.pone.0248006 (PMC7971531; doi:10.1371/journal.pone.0248006)
Supplement: S1 Table — (DOCX) [file pone.0248006.s010.docx]

|  |  |  | MNI Coordinates | | |  |  |
| --- | --- | --- | --- | --- | --- | --- | --- |
| Name of Region | Brodmann Area | Voxels | x | y | z | *t*(29) | *p-value*  (p < .05; FWE-corrected) |
| Decision (C) |  |  |  |  |  |  |  |
| R dorsolateral PFC | 46 | 33 | 48 | 41 | 19 | 7.15 | .001 |
| R ventrolateral PFC | 45 | 25 | 45 | 29 | 28 | 7.93 | .001 |
| R inf parietal lobule | 40 | 37 | 48 | -40 | 46 | 7.72 | .001 |
| L inf parietal lobule | 40 | 42 | -42 | -37 | 37 | 6.66 | .01 |
| R sup parietal lobule | 7 | 30 | 27 | -58 | 40 | 7.08 | .001 |
| L sup parietal lobule | 7 | 32 | -24 | -58 | 49 | 6.77 | .01 |
| L calcarine | 17 | 1594 | -12 | -91 | -2 | 9.93 | .001 |
| Decision to (D) |  |  |  |  |  |  |  |
| R dorsolateral PFC | 9 | 438 | 42 | 29 | 34 | 10.38 | .001 |
| L ventrolateral PFC | 44 | 161 | -51 | 11 | 25 | 8.59 | .001 |
| Ant midcingulate | 32 | 19 | -3 | 29 | 34 | 6.91 | .01 |
| L temporoparietal junction | 40 | 387 | -39 | -49 | 55 | 10.85 | .001 |
| R inf parietal lobule | 40 | 184 | 36 | -49 | 43 | 8.60 | .001 |
| L sup parietal lobule | 7 | 241 | -27 | -61 | 49 | 10.16 | .001 |
| R sup parietal lobule | 7 | 150 | 27 | -64 | 43 | 8.33 | .001 |
| Precuneus | 7 | 212 | 3 | -73 | 46 | 8.10 | .001 |
| L hippocampus |  | 29 | -18 | -31 | -5 | 9.18 | .001 |
| R hippocampus |  | 23 | 27 | -31 | -5 | 9.14 | .001 |
| R cuneus | 17 | 2828 | 15 | -94 | 7 | 13.37 | .001 |

*Note:* *t*(29) = 6.05, *p* < .05; FWE-corrected, *k* > 10
