# Supplementary material for: The Prisoner’s Dilemma paradigm provides a neurobiological framework for the social decision cascade
Source: PLoS One. 2021 Mar 18;16(3):e0248006. doi: 10.1371/journal.pone.0248006 (PMC7971531; doi:10.1371/journal.pone.0248006)
Supplement: S2 Table — (DOCX) [file pone.0248006.s011.docx]

|  |  |  | MNI Coordinates | | |  |  |
| --- | --- | --- | --- | --- | --- | --- | --- |
| Name of Region | Brodmann Area | Voxels | x | y | z | *t*(29) |  |
| Anticipation (C) |  |  |  |  |  |  |  |
| L sup occipital lobe | 17 | 206 | -12 | -94 | 4 | 5.22 | .001 |
| R mid occipital lobe | 18 | 229 | 24 | -91 | 7 | 4.93 | .001 |
| Anticipation (D) |  |  |  |  |  |  | .001 |
| Dorsomedial PFC | 9 | 290 | 6 | 41 | 46 | 5.52 | .001 |
| R ventrolateral PFC | 44 | 119 | 48 | 26 | 28 | 6.04 | .001 |
| L ventrolateral PFC | 44 | 144 | -39 | 20 | 31 | 5.01 | .001 |
| L temporoparietal junction | 40 | 220 | -36 | -46 | 37 | 4.25 | .03 |
| R temporoparietal junction | 40 | 153 | 30 | -62 | 40 | 5.30 | .001 |
| R sup parietal lobule | 7 | 112 | 27 | -64 | 52 | 4.64 | .03 |
| R mid temporal gyrus | 21 | 106 | 57 | -31 | -8 | 6.08 | .001 |
| R calcarine | 17 | 123 | 12 | -97 | 4 | 7.28 | .001 |
| L mid occipital lobe | 7 | 136 | -30 | -58 | 37 | 5.50 | .001 |

*Note:* All results were thresholded at *t*(29)=3.41, *p* < .001 uncorrected voxel-wise threshold; FWE-corrected cluster-wise threshold determined by SPM12.
