# Supplementary material for: The Prisoner’s Dilemma paradigm provides a neurobiological framework for the social decision cascade
Source: PLoS One. 2021 Mar 18;16(3):e0248006. doi: 10.1371/journal.pone.0248006 (PMC7971531; doi:10.1371/journal.pone.0248006)
Supplement: S3 Table — (DOCX) [file pone.0248006.s012.docx]

|  |  |  | MNI Coordinates | | |  |  |
| --- | --- | --- | --- | --- | --- | --- | --- |
| Name of Region | Brodmann Area | Voxels | x | y | z | *t*(29) | *p-value*  (p < .05; FWE-corrected) |
| Reciprocated (CC+DD) |  |  |  |  |  |  |  |
| R dorsolateral PFC | 9 | 47 | 42 | 11 | 40 | 7.30 | .001 |
| L inf parietal lobule | 40 | 135 | -45 | -46 | 43 | 6.85 | .001 |
| R inf parietal lobule | 40 | 90 | 33 | -61 | 40 | 9.91 | .001 |
| R mid occipital lobe | 7 | 115 | 33 | -61 | 40 | 9.91 | .001 |
| L mid occipital lobe | 18 | 171 | -18 | -91 | 1 | 9.40 | .001 |
| Unreciprocated (CD+DC) |  |  |  |  |  |  | .001 |
| Dorsomedial PFC | 9 | 199 | 12 | 44 | 40 | 8.04 | .001 |
| R dorsolateral PFC | 9 | 384 | 36 | 17 | 49 | 8.88 | .001 |
| L ventrolateral PFC | 44 | 239 | -45 | 26 | 34 | 8.68 | .001 |
| R ventrolateral PFC | 48 | 140 | 48 | 14 | 25 | 7.25 | .001 |
| L anterior PFC | 10 | 21 | -33 | 59 | 4 | 7.20 | .001 |
| L temporoparietal junction | 40 | 267 | -36 | -58 | 49 | 7.70 | .001 |
| L sup parietal lobule | 7 | 112 | 30 | -67 | 52 | 8.36 | .001 |
| R sup parietal lobule | 7 | 93 | 42 | -55 | 55 | 7.67 | .001 |
| Precuneus | 7 | 49 | 9 | -70 | 49 | 7.53 | .001 |
| R inf temporal gyrus | 37 | 65 | 60 | -46 | -8 | 8.67 | .001 |
| L mid occipital lobe | 17 | 1155 | -15 | -94 | -2 | 8.68 | .001 |
| Co-Player Cooperation (CC+DC) |  |  |  |  |  |  | .001 |
| R dorsolateral PFC | 46 | 48 | 33 | 59 | 19 | 7.76 | .001 |
| R ventrolateral PFC | 45 | 29 | 45 | 47 | 10 | 7.10 | .001 |
| R inf parietal lobule | 7 | 142 | 30 | -61 | 43 | 7.97 | .001 |
| L inf parietal lobule | 40 | 42 | -33 | -61 | 49 | 7.03 | .001 |
| L mid occipital lobe | 18 | 734 | 21 | -94 | 13 | 8.26 | .001 |
| Co-Player Defection (CD+DD) |  |  |  |  |  |  | .001 |
| R dorsolateral PFC | 46 | 340 | 36 | 17 | 43 | 8.27 | .001 |
| L ant PFC | 10 | 182 | -42 | 53 | 4 | 7.37 | .001 |
| L ventrolateral PFC | 44 | 93 | -36 | 17 | 31 | 8.69 | .001 |
| Ant midcingulate | 32 | 94 | 3 | 26 | 40 | 8.16 | .001 |
| L temporoparietal junction | 40 | 305 | -45 | -49 | 43 | 8.08 | .001 |
| R temporoparietal junction | 40 | 166 | 36 | -61 | 46 | 9.38 | .001 |
| L sup parietal lobule | 7 | 151 | -30 | -64 | 46 | 9.05 | .001 |
| R sup parietal lobule | 7 | 113 | 24 | -67 | 49 | 7.02 | .001 |
| Precuneus | 7 | 67 | 9 | -70 | 52 | 8.49 | .001 |
| R hippocampus |  | 28 | 24 | -28 | -2 | 8.35 | .001 |
| L mid occipital lobe | 18 | 1265 | -15 | -94 | -2 | 8.32 | .001 |

*Note:* *t*(29) = 6.05, *p* < .05; FWE-corrected, *k* > 10
