# Supplementary material for: The Prisoner’s Dilemma paradigm provides a neurobiological framework for the social decision cascade
Source: PLoS One. 2021 Mar 18;16(3):e0248006. doi: 10.1371/journal.pone.0248006 (PMC7971531; doi:10.1371/journal.pone.0248006)
Supplement: S4 Table — (DOCX) [file pone.0248006.s013.docx]

|  |  |  | MNI Coordinates | | |  |  |
| --- | --- | --- | --- | --- | --- | --- | --- |
| Name of Region | Brodmann Area | Voxels | x | y | z | *t*(29) | *p-*value  *(p* < .001;  Clusterwise-FDR corrected) |
| Decision (C) > Decision (D) | | | | | | |  |
| No suprathreshold voxels | | | | | | |  |
| Decision (D) > Decision (C) | | | | | | |  |
| R lingual gyrus | 18 | 136 | 15 | -70 | -8 | 6.05 | .001 |
| Anticipation (C) > (D) | | | | | | |  |
| No suprathreshold voxels | | | | | | |  |
| Anticipation (D) > (C) | | | | | | |  |
| No suprathreshold voxels | | | | | | |  |
| Reciprocated > Unreciprocated | | | | | | |  |
| R postcentral gyrus | 3 | 136 | 42 | -28 | 55 | 5.61 | .001 |
| L post midcingulate | 23 | 54 | -9 | -25 | 52 | 4.98 | .02 |
| L postcentral gyrus | 2 | 55 | -21 | -40 | 61 | 4.18 | .02 |
| Unreciprocated >Reciprocated | | | | | | |  |
| Dorsomedial PFC | 32 | 149 | 6 | 53 | 16 | 4.37 | .001 |
| R ant cingulate | 24 | 114 | 6 | 32 | 13 | 4.86 | .001 |
| L medial OFC | 11 | 51 | -12 | 50 | -11 | 4.55 | .04 |
| R cerebellum crus 2 |  | 60 | 27 | -70 | -38 | 4.63 | .03 |
|  |  |  |  |  |  |  |  |
| Co-Player Cooperation > Defection | | | | | | |  |
| No suprathreshold voxels | | | | | | |  |
| Co-Player Defection > Cooperation | | | | | | |  |
| No suprathreshold voxels | | | | | | |  |
| Decision > Feedback** | | | | | | |  |
| L ventrolateral PFC | 48 | 17 | -42 | 32 | 22 | 6.92 | .001 |
| R ventrolateral PFC | 45 | 15 | 39 | 29 | 31 | 7.06 | .001 |
| L inf parietal lobule | 40 | 261 | -45 | -34 | 40 | 7.95 | .001 |
| R inf parietal lobule | 40 | 94 | 27 | -52 | 43 | 6.90 | .001 |
| L sup parietal lobule | 7 | 128 | -24 | -58 | 52 | 7.80 | .001 |
| L temporal pole | 38 | 57 | -54 | 14 | -8 | 9.98 | .001 |
| R hippocampus | 27 | 34 | 21 | -31 | -2 | 7.27 | .001 |
| L hippocampus | 37 | 29 | -24 | -31 | -2 | 7.27 | .001 |
| Occipital lobe/Cuneus | 17 | 3442 | -12 | -94 | 1 | 11.36 | .001 |
| Feedback > Decision | | | | | | |  |
| No suprathreshold voxels | | | | | | |  |
| Decision > Anticipation** | | | | | | |  |
| L dorsolateral PFC | 46 | 105 | -33 | 53 | 16 | 7.86 | .001 |
| R dorsolateral PFC | 46 | 176 | 33 | 50 | 25 | 7.54 | .001 |
| L ventrolateral PFC | 48 | 129 | -39 | 32 | 19 | 9.13 | .001 |
| R ventrolateral PFC | 45 | 39 | 45 | 29 | 28 | 8.48 | .001 |
| L temporoparietal junction | 40 | 379 | -42 | -34 | 40 | 8.42 | .001 |
| R inf parietal lobule | 40 | 106 | 45 | -40 | 49 | 7.60 | .001 |
| L sup parietal lobule | 7 | 186 | -27 | -61 | 52 | 8.39 | .001 |
| R sup parietal lobule | 7 | 119 | 39 | -52 | 55 | 7.19 | .001 |
| L temporal pole | 38 | 66 | -51 | 14 | -5 | 9.92 | .001 |
| L precuneus | 7 | 11 | 0 | -73 | 52 | 6.72 | .01 |
| R hippocampus | 27 | 63 | 24 | -31 | -2 | 8.64 | .001 |
| L hippocampus | 37 | 50 | -24 | -31 | 1 | 8.48 | .001 |
| L calcarine | 18 | 2581 | -12 | -94 | -2 | 10.83 | .001 |
| Anticipation > Decision | | | | | | |  |
| No suprathreshold voxels |  |  |  |  |  |  |  |
| Feedback > Anticipation** | | | | | | |  |
| R dorsolateral PFC | 46 | 254 | 33 | 47 | 28 | 8.35 | .001 |
| L dorsolateral PFC | 46 | 70 | -30 | 53 | 19 | 7.74 | .001 |
| L ventrolateral PFC | 48 | 112 | -45 | 17 | 31 | 9.04 | .001 |
| R ventrolateral PFC | 44 | 109 | 42 | 11 | 37 | 9.03 | .001 |
| Ant midcingulate | 32 | 194 | -3 | 11 | 49 | 8.61 | .001 |
| L temporoparietal junction | 40 | 251 | -36 | -58 | 55 | 7.97 | .001 |
| R temporoparietal junction | 40 | 90 | 39 | -58 | 46 | 7.81 | .001 |
| L sup parietal lobule | 7 | 162 | -24 | -64 | 52 | 8.81 | .001 |
| Precuneus | 7 | 54 | -6 | -70 | 49 | 6.58 | .01 |
| R hippocampus | 37 | 23 | 27 | -28 | -2 | 7.74 | .001 |
| Occipital lobe/calcarine | 18 | 2735 | -15 | -94 | -2 | 10.70 | .001 |

*Note:* *t*(29)=3.38, *p* < .001 uncorrected voxel-wise threshold; FWE-corrected cluster-wise threshold determined by SPM12.

**Contrast thresholded at [*t(*29)=6.02, *p* < .05 FWE-corrected voxel-wise threshold]
