# Supplementary material for: The Prisoner’s Dilemma paradigm provides a neurobiological framework for the social decision cascade
Source: PLoS One. 2021 Mar 18;16(3):e0248006. doi: 10.1371/journal.pone.0248006 (PMC7971531; doi:10.1371/journal.pone.0248006)
Supplement: S5 Table — (DOCX) [file pone.0248006.s014.docx]

|  |  |  | MNI Coordinates | | |  |  |  |  |  |  |  |  |
| --- | --- | --- | --- | --- | --- | --- | --- | --- | --- | --- | --- | --- | --- |
| Name of Region | Brodmann Area | Voxels | x | y | z | *t*(29) | *p-*value  (*p* < .05; FWE-corrected) |  |  |  |  |  |  |
| Decision Cooperate | | | | | | |  |  |  |  |  |  |  |
| R dlPFC | 46 | 46 | 42 | 56 | 10 | 7.56 | .001 |  |  |  |  |  |  |
| R vlPFC | 45 | 171 | 45 | 44 | 25 | 9.01 | .001 |  |  |  |  |  |  |
| L vlPFC | 48 | 67 | -39 | 20 | 28 | 7.91 | .001 |  |  |  |  |  |  |
| R lateral OFC | 11 | 18 | 27 | 47 | -14 | 8.03 | .001 |  |  |  |  |  |  |
| L lateral OFC | 10 | 49 | -39 | 59 | -2 | 7.70 | .001 |  |  |  |  |  |  |
| R anterior midcingulate | 32 | 20 | 6 | 29 | 34 | 7.09 | .001 |  |  |  |  |  |  |
| L sup parietal lobule | 7 | 238 | -30 | -52 | 49 | 9.34 | .001 |  |  |  |  |  |  |
| R sup parietal lobule | 7 | 195 | 36 | -58 | 55 | 8.98 | .001 |  |  |  |  |  |  |
| L temporoparietal junction | 40 | 475 | -39 | -49 | 43 | 11.46 | .001 |  |  |  |  |  |  |
| R temporoparietal junction | 40 | 201 | 36 | -43 | 40 | 8.76 | .001 |  |  |  |  |  |  |
| L anterior insula | 48 | 17 | -30 | 14 | -2 | 6.57 | .01 |  |  |  |  |  |  |
| L hippocampus | 27 | 50 | -24 | -31 | 4 | 8.66 | .001 |  |  |  |  |  |  |
| R hippocampus | 37 | 35 | 24 | -28 | -2 | 8.24 | .001 |  |  |  |  |  |  |
| R precentral gyrus | 6 | 109 | 27 | 8 | 61 | 8.51 | .001 |  |  |  |  |  |  |
| Occipital lobe, calcarine | 18 | 3004 | -21 | -94 | 4 | 12.85 | .001 |  |  |  |  |  |  |
| Decision Defect | | | | | | |  |  |  |  |  |  |  |
| L vlPFC | 45 | 90 | -48 | 38 | 19 | 7.43 | .001 |  |  |  |  |  |  |
| R anterior PFC | 10 | 11 | 27 | 62 | 16 | 6.55 | .01 |  |  |  |  |  |  |
| L sup parietal lobule | 7 | 211 | -27 | -58 | 48 | 12.11 | .001 |  |  |  |  |  |  |
| R sup parietal lobule | 7 | 132 | 15 | -73 | 49 | 8.31 | .001 |  |  |  |  |  |  |
| L temporoparietal junction | 40 | 268 | -54 | -34 | 49 | 7.37 | .001 |  |  |  |  |  |  |
| R temporoparietal junction | 40 | 71 | 27 | -61 | 46 | 12.20 | .001 |  |  |  |  |  |  |
| R hippocampus | 37 | 39 | 21 | -31 | -2 | 9.31 | .001 |  |  |  |  |  |  |
| L hippocampus | 27 | 32 | -21 | -31 | -2 | 8.28 | .001 |  |  |  |  |  |  |
| Occipital lobe | 18 | 2781 | 36 | -88 | 4 | 13.89 | .001 |  |  |  |  |  |  |

*Note:* *t*(29)=6.04, *p* < .05; FWE-corrected, *k* > 10
