# Supplementary material for: The Prisoner’s Dilemma paradigm provides a neurobiological framework for the social decision cascade
Source: PLoS One. 2021 Mar 18;16(3):e0248006. doi: 10.1371/journal.pone.0248006 (PMC7971531; doi:10.1371/journal.pone.0248006)
Supplement: S6 Table — (DOCX) [file pone.0248006.s015.docx]

|  |  |  | MNI Coordinates | | |  |  |
| --- | --- | --- | --- | --- | --- | --- | --- |
| Name of Region | Brodmann Area | Voxels | x | y | z | *t*(29) | *p-*value  *(p* < .001;  Clusterwise-FDR corrected) |
| Anticipation Cooperate |  |  |  |  |  |  |  |
| Occipital lobe, cuneus | 17 | 99 | 12 | -100 | 10 | 4.65 |  |
| Anticipation Defect |  |  |  |  |  |  |  |
| dmPFC/aMCC | 8 | 388 | 6 | 44 | 28 | 5.68 | .001 |
| L dlPFC | 46 | 111 | -45 | 50 | 1 | 6.11 | .001 |
| R dlPFC | 46 | 46 | 48 | 50 | -8 | 4.18 | .03 |
| R vlPFC | 8 | 283 | 30 | 14 | 49 | 5.46 | .001 |
| L temporoparietal junction | 39 | 187 | -39 | -58 | 49 | 6.32 | .001 |
| R temporoparietal junction | 39 | 190 | 48 | -58 | 40 | 5.30 | .001 |
| R sup parietal lobule | 7 | 43 | 30 | -64 | 43 | 4.60 | .03 |
| Precuneus | 7 | 56 | 0 | -79 | 46 | 5.00 | .001 |
| R sup occipital lobe | 18 | 576 | 21 | -94 | 7 | 6.30 | .001 |

*Note:* All results were thresholded at *t*(29)=3.41, *p* < .001 uncorrected voxel-wise threshold; FDR-corrected cluster-wise threshold determined by SPM12.
