# Supplementary material for: The Prisoner’s Dilemma paradigm provides a neurobiological framework for the social decision cascade
Source: PLoS One. 2021 Mar 18;16(3):e0248006. doi: 10.1371/journal.pone.0248006 (PMC7971531; doi:10.1371/journal.pone.0248006)
Supplement: S7 Table — (DOCX) [file pone.0248006.s016.docx]

|  |  |  | MNI Coordinates | | |  |  |  |  |
| --- | --- | --- | --- | --- | --- | --- | --- | --- | --- |
| Name of Region | Brodmann Area | Voxels | x | y | z | *t*(29) | *p-*value  (*p* < .05; FWE-corrected) | |  |
| Reciprocated Feedback |  |  |  |  |  |  |  |  |  |
| dmPFC/aMCC | 32 | 207 | -3 | 20 | 40 | 8.62 | .001 |  |  |
| R dlPFC | 9 | 470 | 45 | 29 | 34 | 8.73 | .001 |  |  |
| L vlPFC | 48 | 69 | -42 | 23 | 28 | 7.13 | .001 |  | |
| R vlPFC | 45 | 64 | 51 | 32 | 28 | 8.33 | .001 |  |  |
| R lateral OFC | 11 | 102 | 30 | 44 | -11 | 10.14 | .001 |  |  |
| L lateral OFC | 46 | 54 | -42 | 50 | -2 | 8.32 | .001 |  |  |
| L temporoparietal junction | 40 | 244 | -36 | -46 | 37 | 8.83 | .001 |  |  |
| R temporoparietal junction | 40 | 171 | 42 | -55 | 49 | 8.57 | .001 |  |  |
| L sup parietal lobule | 7 | 170 | -24 | -70 | 46 | 9.02 | .001 |  |  |
| R sup parietal lobule | 7 | 87 | 30 | -67 | 43 | 9.61 | .001 |  |  |
| Precuneus | 7 | 211 | 3 | -73 | 49 | 8.76 | .001 |  |  |
| R anterior insula | 47 | 68 | 39 | 17 | -8 | 7.79 | .001 |  |  |
| L anterior insula | 48 | 28 | -33 | 23 | -5 | 6.18 | .001 |  |  |
| R hippocampus | 27 | 35 | 21 | -28 | -2 | 7.72 | .001 |  |  |
| L hippocampus | 37 | 17 | -24 | -31 | 4 | 7.08 | .001 |  |  |
| R putamen |  | 131 | 27 | 14 | -8 | 8.97 | .001 |  |  |
| L putamen |  | 59 | -24 | 17 | -2 | 8.70 | .001 |  |  |
| Occipital lobe, calcarine | 18 | 2395 | -15 | -94 | -2 | 10.60 | .001 |  |  |
| Unreciprocated Feedback |  |  |  |  |  |  | .001 |  |  |
| dmPFC/aMCC | 32 | 39 | 6 | 35 | 40 | 7.80 | .001 |  |  |
| R dlPFC | 9 | 293 | 45 | 50 | 4 | 7.36 | .001 |  |  |
| L vlPFC | 48 | 242 | -42 | 29 | 25 | 8.70 | .001 |  |  |
| R vlPFC | 44 | 329 | 48 | 29 | 31 | 7.94 | .001 |  |  |
| L temporoparietal junction | 40 | 261 | -33 | -52 | 40 | 9.68 | .001 |  |  |
| R temporoparietal junction | 40 | 142 | 45 | -55 | 31 | 7.04 | .001 |  |  |
| L sup parietal lobule | 7 | 120 | -24 | -73 | 49 | 8.04 | .001 |  |  |
| R sup parietal lobule | 7 | 66 | 30 | -67 | 43 | 11.33 | .001 |  |  |
| Precuneus | 7 | 110 | -3 | -73 | 49 | 7.03 | .001 |  |  |
| L temporal pole | 38 | 22 | -33 | 17 | -23 | 7.98 | .001 |  |  |
| R anterior insula | 48 | 50 | 27 | 17 | -14 | 7.67 | .001 |  |  |
| R hippocampus | 27 | 21 | 21 | -31 | -5 | 7.70 | .001 |  |  |
| L hippocampus | 37 | 18 | -24 | -28 | -2 | 7.35 | .001 |  |  |
| Occipital lobe, calcarine | 18 | 2037 | 21 | -94 | 1 | 11.36 | .001 |  |  |
| Feedback Co-Player Cooperation |  |  |  |  |  |  | .001 |  |  |
| dmPFC/aMCC | 32 | 44 | 6 | 35 | 40 | 7.65 | .001 |  |  |
| R dlPFC | 46 | 26 | 45 | 53 | 7 | 7.33 | .001 |  |  |
| L lateral OFC | 46 | 28 | -42 | 50 | -2 | 8.13 | .001 |  |  |
| L vlPFC | 48 | 167 | -39 | 23 | 28 | 9.49 | .001 |  |  |
| R vlPFC | 45 | 294 | 51 | 35 | 28 | 8.97 | .001 |  |  |
| L temporoparietal junction | 40 | 176 | -39 | -58 | 49 | 8.07 | .001 |  |  |
| R temporoparietal junction | 40 | 53 | 39 | -55 | 43 | 7.19 | .001 |  |  |
| L sup parietal lobule | 7 | 97 | -21 | -73 | 49 | 7.33 | .001 |  |  |
| R sup parietal lobule | 7 | 55 | 30 | -67 | 43 | 9.69 | .001 |  |  |
| Precuneus | 7 | 112 | 3 | -73 | 49 | 8.47 | .001 |  |  |
| R hippocampus | 27 | 22 | 21 | -31 | -5 | 7.57 | .001 |  |  |
| Occipital lobe, calcarine | 18 | 2138 | 21 | -94 | 1 | 10.76 | .001 |  |  |
| Feedback Co-Player Defection |  |  |  |  |  |  | .001 |  |  |
| dmPFC/aMCC | 32 | 263 | 6 | 29 | 40 | 8.64 | .001 |  |  |
| R dlPFC | 9 | 527 | 42 | 29 | 37 | 10.68 | .001 |  |  |
| R vlPFC | 45 | 65 | 42 | 29 | 37 | 10.68 | .001 |  |  |
| L vlPFC | 45 | 117 | -45 | 29 | 22 | 8.90 | .001 |  |  |
| L lateral OFC | 46 | 18 | -45 | 50 | -8 | 7.22 | .001 |  |  |
| L temporoparietal junction | 40 | 302 | -39 | -46 | 37 | 10.09 | .001 |  |  |
| R temporoparietal junction | 40 | 235 | 51 | -40 | 43 | 9.56 | .001 |  |  |
| L sup parietal lobule | 7 | 178 | -30 | -64 | 43 | 10.43 | .001 |  |  |
| R sup parietal lobule | 7 | 94 | 30 | -64 | 40 | 11.24 | .001 |  |  |
| Precuneus | 7 | 199 | 9 | -70 | 46 | 7.75 | .001 |  |  |
| R anterior insula | 48 | 191 | 27 | 17 | -11 | 9.17 | .001 |  |  |
| L anterior insula | 47 | 98 | -30 | 17 | -17 | 8.52 | .001 |  |  |
| L hippocampus | 27 | 15 | -18 | -31 | -2 | 7.17 | .001 |  |  |
| R hippocampus | 37 | 20 | 21 | -28 | -5 | 6.56 | .01 |  |  |
| Occipital lobe, calcarine | 18 | 2414 | 21 | -97 | 7 | 11.77 | .001 |  |  |

*Note:* *t*(29)=5.98, *p* < .05 FWE-corrected, *k* > 10
