# Supplementary material for: The Prisoner’s Dilemma paradigm provides a neurobiological framework for the social decision cascade
Source: PLoS One. 2021 Mar 18;16(3):e0248006. doi: 10.1371/journal.pone.0248006 (PMC7971531; doi:10.1371/journal.pone.0248006)
Supplement: S8 Table — (DOCX) [file pone.0248006.s017.docx]

|  |  |  | MNI Coordinates | | |  |  |  |  |  |  |  |  |  |
| --- | --- | --- | --- | --- | --- | --- | --- | --- | --- | --- | --- | --- | --- | --- |
| Name of Region | Brodmann Area | Voxels | x | y | z | *t*(29) | *p-*value  *(p* < .001;  Clusterwise-FDR corrected) | |  |  |  |  |  |  |
| Decision Cooperate>Defect | | | | | | |  |  | |  |  |  |  |  |
| L lingual gyrus | 18 | 46 | -9 | -70 | -8 | 4.63 | .03 |  |  |  |  |  |  |  |
| Decision Defect>Cooperate | | | | | | |  |  |  |  |  |  |  |  |
| No suprathreshold voxels |  |  |  |  |  |  |  |  |  |  |  |  |  |  |
| Anticipation Cooperate>Defect | | | | | | |  |  |  |  |  |  |  |  |
| No suprathreshold voxels |  |  |  |  |  |  |  |  |  |  |  |  |  |  |
| Anticipation Defect>Cooperate | | | | | | |  |  |  |  |  |  |  |  |
| dmPFC | 8 | 47 | 6 | 35 | 49 | 4.06 | .04 |  |  |  |  |  |  |  |
| R dlPFC | 8 | 68 | 24 | 26 | 52 | 4.41 | .03 |  |  |  |  |  |  |  |
| L vlPFC | 44 | 45 | -51 | 11 | 25 | 4.51 | .04 |  |  |  |  |  |  |  |
| R precentral gyrus | 4 | 46 | 39 | -22 | 55 | 4.12 | .04 |  |  |  |  |  |  |  |
| Occipital lobe | 19 | 61 | -42 | -73 | -11 | 4.00 | .03 |  |  |  |  |  |  |  |
| Feedback Recip>Unrecip | | | | | | |  |  |  |  |  |  |  |  |
| No suprathreshold voxels |  |  |  |  |  |  |  |  |  |  |  |  |  |  |
| Feedback Unrecip>Recip | | | | | | |  |  |  |  |  |  |  |  |
| No suprathreshold voxels |  |  |  |  |  |  |  |  |  |  |  |  |  |  |
| Feedback Co-Player Cooperate>Defect | | | | | | |  |  |  |  |  |  |  |  |
| No suprathreshold voxels |  |  |  |  |  |  |  |  |  |  |  |  |  |  |
| Feed Co-Player Defect>Cooperate | | | | | | |  |  |  |  |  |  |  |  |
| R temporoparietal junction | 40 | 258 | 57 | -43 | 40 | 6.47 | .001 |  |  |  |  |  |  |  |
| L anterior insula | 48 | 92 | -27 | 11 | -17 | 5.85 | .01 |  |  |  |  |  |  |  |
| R anterior insula | 47 | 71 | 30 | 20 | -17 | 5.56 | .02 |  |  |  |  |  |  |  |
| Decision>Feedback** | | | | | | |  |  |  |  |  |  |  |  |
| L temporoparietal junction | 40 | 345 | -54 | -31 | 52 | 10.35 | .001 |  |  |  |  |  |  |  |
| L sup parietal lobule | 7 | 183 | -24 | -61 | 49 | 8.88 | .001 |  |  |  |  |  |  |  |
| R sup parietal lobule | 7 | 105 | 27 | -58 | 49 | 8.62 | .001 |  |  |  |  |  |  |  |
| R postcentral gyrus | 2 | 18 | 57 | -25 | 40 | 7.32 | .001 |  |  |  |  |  |  |  |
| L precentral gyrus | 6 | 36 | -48 | 2 | 34 | 7.14 | .001 |  |  |  |  |  |  |  |
| L anterior insula | 48 | 41 | -42 | -1 | 4 | 8.07 | .001 |  |  |  |  |  |  |  |
| R hippocampus | 37 | 44 | 21 | -31 | -2 | 9.61 | .001 |  |  |  |  |  |  |  |
| L hippocampus | 27 | 39 | -21 | -31 | -2 | 7.83 | .001 |  |  |  |  |  |  |  |
| Occipital lobe | 17 | 2885 | -9 | -100 | 4 | 13.72 | .001 |  |  |  |  |  |  |  |
| Decision>Anticipation** | | | | | | |  |  |  |  |  |  |  |  |
| R dlPFC | 46 | 171 | 42 | 41 | 34 | 7.55 | .001 |  |  |  |  |  |  |  |
| L vlPFC | 44 | 287 | -51 | 23 | 34 | 8.44 | .001 |  |  |  |  |  |  |  |
| R vlPFC | 44 | 22 | 48 | 5 | 25 | 7.13 | .001 |  |  |  |  |  |  |  |
| L temporoparietal junction | 40 | 482 | -51 | -34 | 49 | 10.53 | .001 |  |  |  |  |  |  |  |
| R temporoparietal junction | 40 | 161 | 33 | -46 | 43 | 9.40 | .001 |  |  |  |  |  |  |  |
| L sup parietal lobule | 7 | 272 | -27 | -58 | 49 | 12.43 | .001 |  |  |  |  |  |  |  |
| R sup parietal lobule | 7 | 191 | 27 | -58 | 43 | 13.58 | .001 |  |  |  |  |  |  |  |
| Precuneus | 7 | 245 | -6 | -76 | 49 | 8.71 | .001 |  |  |  |  |  |  |  |
| L anterior insula | 48 | 58 | -45 | 14 | 4 | 7.41 | .001 |  |  |  |  |  |  |  |
| L hippocampus | 27 | 78 | -24 | -31 | 1 | 10.06 | .001 |  |  |  |  |  |  |  |
| R hippocampus | 37 | 74 | 21 | -28 | -2 | 9.89 | .001 |  |  |  |  |  |  |  |
| Occipital lobe | 17 | 3096 | 15 | -97 | 13 | 13.90 | .001 |  |  |  |  |  |  |  |
| Feedback>Anticipation** | | | | | | |  |  |  |  |  |  |  |  |
| dmPFC/aMCC | 32 | 298 | -3 | 26 | 40 | 9.76 | .001 |  |  |  |  |  |  |  |
| L dlPFC | 46 | 87 | -30 | 56 | 13 | 7.61 | .001 |  |  |  |  |  |  |  |
| R dlPFC | 46 | 677 | 24 | 53 | 28 | 8.64 | .001 |  |  |  |  |  |  |  |
| R anterior PFC | 10 | 14 | 6 | 62 | 28 | 9.41 | .001 |  |  |  |  |  |  |  |
| R vlPFC | 45 | 76 | 45 | 41 | 31 | 9.91 | .001 |  |  |  |  |  |  |  |
| L vlPFC | 45 | 189 | -42 | 35 | 25 | 8.95 | .001 |  |  |  |  |  |  |  |
| R lateral OFC | 47 | 36 | 45 | 47 | -14 | 7.48 | .001 |  |  |  |  |  |  |  |
| L temporoparietal junction | 40 | 371 | -48 | -43 | 46 | 10.19 | .001 |  |  |  |  |  |  |  |
| R temporoparietal junction | 40 | 198 | 39 | -46 | 40 | 9.48 | .001 |  |  |  |  |  |  |  |
| R sup parietal lobule | 7 | 114 | 36 | -61 | 40 | 14.03 | .001 |  |  |  |  |  |  |  |
| L sup parietal lobule | 7 | 197 | -39 | -61 | 49 | 10.24 | .001 |  |  |  |  |  |  |  |
| Precuneus | 7 | 251 | 3 | -67 | 52 | 7.21 | .001 |  |  |  |  |  |  |  |
| R temporal pole | 38 | 15 | 48 | 20 | -14 | 7.14 | .001 |  |  |  |  |  |  |  |
| R anterior insula | 47 | 15 | 30 | 23 | -17 | 6.84 | .01 |  |  |  |  |  |  |  |
| R hippocampus | 37 | 115 | 21 | -28 | -5 | 10.61 | .001 |  |  |  |  |  |  |  |
| L hippocampus | 27 | 142 | -27 | -31 | 4 | 9.93 | .001 |  |  |  |  |  |  |  |
| Occipital lobe | 18 | 2754 | 21 | -94 | 1 | 12.07 | .001 |  |  |  |  |  |  |  |

*Note:* *t*(29)=3.38, *p* < .001 uncorrected voxel-wise threshold; FWE-corrected cluster-wise threshold determined by SPM12.

**Between-phase contrasts thresholded at [*t(*29)=6.0, *p* < .05 FWE-corrected voxel-wise threshold].
