# Supplementary material for: The Prisoner’s Dilemma paradigm provides a neurobiological framework for the social decision cascade
Source: PLoS One. 2021 Mar 18;16(3):e0248006. doi: 10.1371/journal.pone.0248006 (PMC7971531; doi:10.1371/journal.pone.0248006)
Supplement: S9 Table — (DOCX) [file pone.0248006.s018.docx]

|  |  |  | MNI Coordinates | | |  |  |
| --- | --- | --- | --- | --- | --- | --- | --- |
| Name of Region | Brodmann Area | Voxels | x | y | z | *t*(29) | *p-*value  *(p* < .001;  Clusterwise-FDR corrected) |
| Co-Player Defection (CD+DD)  (Human>Computer) | | | | | | |  |
| L ant insula | 48 | 58 | -27 | 14 | -17 | 5.80 | 0.02 |
| Unreciprocated Cooperation (CD)  (Human>Computer) | | | | | | |  |
| L ant insula | 48 | 44 | -30 | 14 | -11 | 5.81 | 0.02 |
| Mutual Defection (DD)  (Human>Computer) | | | | | | |  |
| No suprathreshold voxels |  |  |  |  |  |  |  |

*Note: t*(29)=3.38, *p* < .001 uncorrected voxel-wise threshold; FWE-corrected cluster-wise threshold determined by SPM12.
